# Editorial: Artificial intelligence solutions for global health and disaster response: challenges and opportunities

**DOI:** 10.3389/fpubh.2024.1439914

**Published:** 2024-08-22

**Authors:** Dmytro Chumachenko, Plinio Pelegrini Morita, Saman Ghaffarian, Tetyana Chumachenko

**Affiliations:** ^1^Mathematical Modelling and Artificial Intelligence Department, National Aerospace University “Kharkiv Aviation Institute”, Kharkiv, Ukraine; ^2^Ubiquitous Health Technologies Lab, University of Waterloo, Waterloo, ON, Canada; ^3^Institute for Risk and Disaster Reduction, University College London, London, United Kingdom; ^4^Epidemiology Department, Kharkiv National Medical University, Kharkiv, Ukraine

**Keywords:** artificial intelligence, public health, global health, disaster response, risk assessment

Artificial intelligence (AI) has emerged as a transformative tool in disaster preparedness and response, promising to reshape how we address some of the most pressing global health challenges. The rapid development of AI technologies offers unprecedented opportunities to enhance early warning systems, optimize resource allocation, and facilitate real-time decision-making processes. This editorial introduces a Research Topic of articles that explore the cutting-edge applications of AI across various domains of global health and disaster response, aiming to provide a comprehensive overview of both the potential and the hurdles associated with these technologies.

This Research Topic gathers insights from leading researchers, practitioners, and policymakers at the forefront of integrating AI into health crisis management. The scope of discussion spans multiple critical areas, including AI-powered surveillance systems, AI-supported decision-making in resource distribution, and the role of AI in managing mental health during crises. Each contribution advances our understanding of AI's capabilities and critically examines the ethical, legal, and social implications of deploying AI in sensitive and high-stakes environments. Through this Research Topic, we aim to highlight the innovative ways in which AI can promote health equity and improve healthcare delivery during disasters while also addressing the significant challenges that must be overcome to harness the full potential of AI in enhancing global health outcomes.

The Research Topic includes original research, systematic reviews, brief research reports, mini reviews, perspective, review, and methods papers. We received 66 submissions, 25 of which, after a careful review process, were accepted for publication in this Research Topic.

The paper by Liu P. et al. explores healthcare professionals' and patients' perceptions and experiences regarding mobile health (mHealth) apps in China. The study identifies the advantages and challenges of using mHealth apps using semistructured interviews. It highlights concerns such as the ease of use, effectiveness, and potential risks associated with these apps. The findings underscore the need for improved safety, regulatory standards, and user-friendly designs to serve older populations better and bridge the gap between healthcare providers and patients. The paper advocates for advancements in mHealth technology to enhance its utility and reliability in improving health outcomes.

The paper by Singh et al. delves into the burgeoning role of blockchain technology within the healthcare sector. It thoroughly examines how blockchain's core features—such as decentralization, immutability, and transparency—can address persistent challenges in healthcare, such as data fragmentation, security concerns, and the lack of interoperability among systems. By proposing a framework for the practical application of blockchain in various healthcare processes, the authors argue that blockchain technology can significantly enhance data security, patient privacy, and the efficiency of healthcare delivery systems. Moreover, the paper discusses the broader implications of blockchain integration into healthcare, including potential ethical and regulatory challenges.

Belachew et al.'s study investigates the adoption and utilization of telemedicine among patients with chronic diseases at the University of Gondar Comprehensive Specialized Hospital. The research uses a cross-sectional study design to capture the perceptions, willingness, and actual use of telemedicine services among these patients. Findings indicate a generally positive perception and willingness to engage with telemedicine despite low usage. This discrepancy highlights the potential for greater implementation of telemedicine, provided that patient awareness and infrastructural support are enhanced.

The paper by Shakhovska et al. investigates micro-stresses' impact on operators' performance within search systems. The study assesses operator behavior under stress by presenting test images to monitor reaction times and decision-making processes utilizing a human-machine interface model. Key findings include the development of a methodology to analyze and cluster operator responses based on their stress resistance. The study offers insights into optimizing the selection and training of operators by recognizing individual differences in stress handling.

The paper by Li et al. introduces a fractal multi-level distribution network (FMDN) model to optimize the delivery of essential supplies during disasters. This model considers factors like road damage and dynamic demand changes at disaster sites to minimize overall operational costs, which include construction, transportation, and penalty costs for unmet demand. The study confirms the model's effectiveness through numerical experiments using LINGO software, demonstrating that it can efficiently manage emergency resource distribution under variable and challenging conditions.

The paper by Pinto et al. examines the influence of online news on public health responses by focusing on the syphilis epidemic in Brazil. The study analyzes the volume and quality of news between 2015 and 2019 and its correlation with syphilis testing rates using text mining techniques. The findings reveal a positive association between high-quality news and increased syphilis testing, emphasizing the role of effective communication in enhancing public health policy actions.

The paper by Zhang et al. examines the influences on Chinese type 2 diabetes patients' readiness to adopt digital disease management apps. Utilizing the Technology Acceptance Model (TAM), Perceived Risk (PR) theory, and eHealth Literacy Theory (E-HLT), the study highlights significant predictors such as perceived usefulness, ease of use, and electronic health literacy. Results indicate these factors substantially impact patients' attitudes toward and intentions to use such applications, providing critical insights for developers and healthcare providers aiming to increase digital health tool adoption.

The paper by Sen et al. addresses the growing relevance of big data analytics in medical research, focusing specifically on text-mining techniques. It discusses various challenges encountered in this field, such as data quality and integration, privacy concerns, and the need for robust analytical tools. The authors propose several recommendations to overcome these hurdles, emphasizing the development of more sophisticated data processing algorithms and implementing stricter data privacy regulations. The paper aims to guide future research and development in efficiently utilizing medical big data for improved healthcare outcomes.

The paper by Towler et al. evaluates the efficacy of Machine-Assisted Topic Analysis (MATA) against traditional human-only thematic analysis. The study, conducted during the COVID-19 pandemic, used a dataset of 1,472 user responses from a behavioral intervention to compare the two methods. The results show that while human analysis provides depth, MATA significantly reduces the time required for data analysis. Both methods produced similar thematic outcomes, demonstrating that MATA is an effective tool for rapid qualitative analysis in public health emergencies.

The paper by Honchar et al. investigates the predictors of long-term health effects in COVID-19 patients post-discharge. The study assesses symptoms, performs echocardiography, and conducts a 6-min walk test both pre-discharge and 1-month post-discharge, utilizing a cohort of 221 hospitalized patients. The presence of post-COVID-19 syndrome (PCS) was then evaluated 3 months after discharge. The research identifies key pre-discharge predictors for PCS, including age, sex, inflammation levels, and oxygen needs. The study successfully developed a neural network-based classification model that predicts the development of PCS with high accuracy, demonstrating a significant step toward optimizing patient care post-COVID-19.

The study by Lihua et al. evaluates the effectiveness of a health self-management intervention tailored for individuals with metabolic syndrome (MS) who were bereaved by the Wenchuan earthquake. This randomized controlled trial utilized a detailed intervention program based on self-management principles, covering diet, exercise, medication adherence, and emotional management. Results showed significant improvements in MS management behaviors and some physiological indicators among the intervention group compared to controls, demonstrating the potential of structured self-management programs in enhancing disease outcomes in post-disaster settings.

The paper by Zhou et al. comprehensively evaluates various AI-based intraocular lens (IOL) power calculation formulas compared to traditional and newer vergence formulas. The study systematically analyzed 12 studies involving 2,430 eyes, utilizing methods such as mean absolute error (MAE), median absolute error (MedAE), and the percentage of eyes with a predictive error within specific diopters focused on highly myopic eyes. The top-performing AI-based formulas identified were XGBoost, Hill-RBF, and Kane, demonstrating superior accuracy over traditional methods for calculating IOL power for highly myopic patients. This paper highlights the potential of AI to enhance the precision of medical predictions in specialized settings.

The study by Ahun et al. investigates Turkish emergency physicians' ethical perspectives on using AI in epidemic triage. The study assesses the attitudes of 167 specialists toward AI's utility in emergency triage, particularly under pandemic conditions, using a detailed survey. The findings reveal a cautious optimism about AI's benefits for patient care and healthcare operations. However, significant concerns remain regarding responsibility for AI-driven decisions and the ethical handling of private patient data. Most respondents acknowledge AI's potential to enhance triage efficiency but emphasize the need for clear accountability and data privacy guidelines.

The paper by Long et al. explores the application of AI to optimize healthcare supply chain modes. It introduces a deep reinforcement learning algorithm to improve decision-making in selecting sustainable and efficient supply chain modes. Through simulation experiments, the study demonstrates that AI can effectively enhance the economic, social, and environmental benefits of healthcare supply chains. The findings suggest AI is a superior healthcare supply chain mode selection method, offering significant improvements over traditional methods.

The paper by Kryvenko et al. addresses the increasing need for effective compression techniques in storing and transferring large dental images. The authors explore lossy compression that retains the visual quality necessary for diagnostic purposes, emphasizing the role of discrete cosine transform-based encoders. They evaluate the performance of different encoders in achieving high compression ratios while ensuring the invisibility of compression artifacts through theoretical approaches and experimental validation with professional dentists. This study is crucial for advancing digital imaging in dental practice and improving telemedicine applications by enabling efficient image data management.

The paper by Zaidan provides a comprehensive analysis of how AI is being integrated into various aspects of global health, addressing significant challenges such as mental health, infectious diseases, cardiovascular diseases, and the impacts of aging. The review explores AI's role in enhancing disease surveillance, diagnosis, treatment modalities, and overall public health strategies. Additionally, it discusses the ethical considerations and the necessity for equitable AI integration in healthcare practices globally, highlighting AI's transformative potential while acknowledging the complexities and responsibilities accompanying its widespread adoption.

The paper by Valeanu et al. examines vaccine misinformation on Twitter, focusing on content written in Romanian. Using a dataset of 1,400 tweets, researchers manually classified each tweet as true, neutral, or fake information. They employed machine learning algorithms to predict the classification of tweets, finding that misinformation tends to be more frequently liked and shared. This study highlights the significant role of AI in distinguishing between valid and false health information online, aiming to support public health efforts by mitigating the spread of vaccine misinformation.

The paper by Yoon et al. examines the application of virtual reality (VR) and virtual try-on technologies to address privacy concerns in home-based fitness training. It introduces methods to anonymize participants' appearances and environments during video-based exercise sessions, enhancing user comfort and motivation. The study demonstrates the effectiveness of these technologies through a user study, highlighting their potential to enhance privacy, self-confidence, and coaching satisfaction. However, no significant differences in coaching satisfaction were noted. This innovative approach proposes a model for future remote fitness training that prioritizes user privacy and engagement.

The study by Roche et al. investigates the accuracy of AI in reading and interpreting HIV self-test results. The study, conducted in Kisumu, Kenya, involved participants using blood-based HIV self-tests in private pharmacies, with results interpreted by clients, pharmacy providers, and an AI algorithm. The AI algorithm demonstrated high sensitivity and specificity, showing promise as a quality assurance tool in HIV testing, comparing these interpretations to an expert panel's readings. The findings suggest that AI could enhance accuracy and reliability in interpreting HIV self-tests in real-world settings.

The paper by Tucker and Lorig explores the integration of agent-based social simulations (ABSS) with the everyday digital health perspective to inform policy during health crises like COVID-19. ABSS models the interactions of intelligent agents to simulate complex societal responses to health policies, serving as a virtual testbed for scenario testing without risking real-world consequences. The paper highlights challenges such as the need for valuable scenario definition and appropriate data availability, suggesting that incorporating everyday digital health insights can enhance ABSS's effectiveness in crisis management. This approach emphasizes the importance of understanding digital health technology adoption and its varied impacts across different population segments to improve the accuracy and relevance of health crisis simulations.

The paper by Benboujja et al. addresses the significant challenge of language barriers in pediatric healthcare. It presents a multilingual, AI-assisted curriculum designed to improve global healthcare education. The curriculum includes video modules in multiple languages tailored for diverse healthcare settings using generative language models, enhancing comprehension and accessibility for non-English speaking healthcare providers and caregivers. This innovative approach ensures that essential pediatric care knowledge is universally accessible, supporting the World Health Organization's advocacy for digitally enabled healthcare education.

The paper by Alotaibi et al. examines the application of AI, specifically ChatGPT, in the management of ocular cancer. It reviews existing literature to address the types of ocular cancer, the associated challenges, and how ChatGPT can assist in overcoming these barriers. The review underscores the limited awareness and healthcare access, financial constraints, and infrastructure deficiencies as significant hurdles in effective ocular cancer management. The paper discusses the prospective benefits of integrating ChatGPT to enhance diagnostic accuracy, patient education, and treatment planning while highlighting the necessity for further research to optimize AI applications in healthcare.

The paper by Moskalenko and Kharchenko discusses enhancing the robustness of medical AI systems against disturbances like adversarial attacks, fault injections, and data drift. It introduces a modified machine learning operations (MLOps) framework incorporating resilience optimization, predictive uncertainty calibration, and graceful degradation. The study demonstrates that adding these resilience mechanisms improves the system's ability to handle disruptions, particularly in medical image recognition, using datasets like DermaMNIST, BloodMNIST, and PathMNIST. The findings suggest that AI systems can be more reliable and trustworthy in critical healthcare applications by integrating these resilience-focused adaptations.

The paper by Miao et al. introduces a machine learning model for the early identification of elevated arterial stiffness (EAS) using easily accessible clinical and questionnaire data from 77,134 participants. The study demonstrates the effectiveness of the XGBoost algorithm, which outperformed other models in accuracy and predictive capability, utilizing advanced feature selection and model training methods. The research emphasizes the potential of this cost-effective model to significantly enhance the screening processes for arterial aging, making it accessible for broad clinical application.

The paper by Liu X.-d. et al. focuses on enhancing the predictive accuracy of epidemic time series data by incorporating Gated Recurrent Units (GRU) into Graph Neural Networks (GNN). This integration, referred to as GRGNN, is evaluated using datasets including COVID-19 cases from African and European countries and a chickenpox dataset from Hungarian regions. The study demonstrates that GRGNN consistently outperforms traditional models in prediction accuracy, validating its effectiveness in epidemic forecasting and highlighting its potential for early warning systems in public health.

The Research Topic of articles within this Research Topic underscores AI's vast potential and multifaceted challenges in enhancing disaster preparedness and response. From improving early warning systems to ensuring health equity in crises, the insights provided by these papers highlight both the transformative capabilities and the ethical considerations necessary for the responsible deployment of AI technologies. As we move forward, the continued collaboration between researchers, practitioners, and policymakers will be crucial in harnessing AI's power to respond to, anticipate, and mitigate global health emergencies.

## Author contributions

DC: Writing – original draft, Writing – review & editing. PM: Writing – original draft, Writing – review & editing. SG: Writing – original draft, Writing – review & editing. TC: Writing – original draft, Writing – review & editing.

